# Treatment outcomes of pulmonary TB in adults in Indonesia

**DOI:** 10.5588/ijtldopen.24.0482

**Published:** 2025-03-12

**Authors:** R.I. Sugiyono, A.M. Naysilla, N.H. Susanto, D. Handayani, E. Burhan, A. Karuniawati, T. Kusmiati, B.H. Wibisono, B.S. Riyanto, I.G.K. Sajinadiyasa, I. Djaharuddin, B.Y.M. Sinaga, R.D. Dewantara, M. Karyana, H. Kosasih, C.J. Liang, R. Ridzon, A.T. Neal, R.Y. Chen

**Affiliations:** ^1^ Indonesia Research Partnership on Infectious Diseases (INA-RESPOND), Jakarta, Indonesia;; ^2^ Department of Pulmonology and Respiratory Medicine, Faculty of Medicine, Universitas Indonesia, Persahabatan Hospital, Jakarta, Indonesia;; ^3^ Department of Microbiology, Faculty of Medicine, Universitas Indonesia, Dr. Cipto Mangunkusumo Hospital, Jakarta, Indonesia;; ^4^ Department of Pulmonology and Respiratory Medicine, Faculty of Medicine, Universitas Airlangga, Dr. Soetomo General Hospital, Surabaya, Indonesia;; ^5^ Division Pulmonology and Critical Care, Department of Internal Medicine, Kariadi Hospital, Semarang, Indonesia;; ^6^ Division of Pulmonology, Department of Internal Medicine Sardjito Hospital, Yogyakarta, Indonesia;; ^7^ Pulmonology Division of Internal Medicine Department, Prof. Dr. I.G.N.G. Ngoerah Hospital, Denpasar, Indonesia;; ^8^ Department of Pulmonology and Respiratory Medicine, Faculty of Medicine, Hasanuddin University, Wahidin Sudirohusodo Hospital, Makassar, Indonesia;; ^9^ Department of Pulmonology and Respiratory Medicine, Adam Malik Hospital, Faculty of Medicine, Universitas Sumatera Utara, Medan, Indonesia;; ^10^ Health Policy Agency, Ministry of Health, Jakarta, Indonesia;; ^11^ Biostatistics Research Branch, Division of Clinical Research, National Institute of Allergy and Infectious Diseases, National Institutes of Health, Bethesda, MD, USA;; ^12^ Collaborative Clinical Research Branch, Division of Clinical Research, National Institute of Allergy and Infectious Diseases, National Institutes of Health, Bethesda, MD, USA.

**Keywords:** drug-susceptible tuberculosis, multidrug resistance, cure, failure, death

## Abstract

**BACKGROUND:**

Achieving the goal of Ending TB requires a treatment success rate of ≥90%, a challenging target for Indonesia. To understand outcomes and associated factors for unfavourable outcomes, we analysed prospective multicentre study data from 2017 to 2020 involving drug-susceptible TB (DS-TB) and multidrug-resistant TB (MDR-TB) treatment adult patients.

**METHODS:**

This study focused on bacteriologically confirmed participants based on Xpert MTB/RIF or culture results. We analysed participants with available treatment outcomes — cured, completed, failed, dead, and lost to follow-up (LTFU) — excluding withdrawn or transferred. Univariable and multivariable logistic regression analyses identified factors associated with unfavourable outcomes.

**RESULTS:**

Among 328 bacteriologically confirmed participants with available outcomes, the overall treatment success was 72.3% (DS-TB: 81.6% and MDR-TB: 60.1%). Unfavourable outcomes were 27.7%, with 3.6% failures, 9.5% deaths, and 14.6% LTFUs. Associated factors for unfavourable outcomes included age ≥50 years (aOR 2.54, 95% CI 1.11–5.95; *P* = 0.029); being underweight (aOR 1.93, 95% CI 1.05–3.61; *P* = 0.037); having baseline acid-fast bacilli smear of scanty/+1 (aOR 3.77, 95% CI 1.41–11.65; *P* = 0.013) or +2/+3 (aOR 3.34, 95% CI 1.31–9.83; *P* = 0.017); and being treated with MDR-TB regimen (aOR 2.03, 95% CI 1.05–3.96; *P* = 0.036).

**CONCLUSION:**

Strategies to improve outcomes include tailored care for older adults, nutritional support, treatment monitoring, and enhanced MDR-TB management.

Indonesia is classified by the WHO as a high TB burden country, with a national TB incidence of 385/100,000 population in 2023.^1^ Additionally, Indonesia is among the ten countries with the highest burden of MDR/rifampicin-resistant TB (MDR/RR-TB): 2.4% in new cases and 17.8% in previously treated cases.^1,2^ The Indonesian government has expanded access to rapid molecular diagnostic tests and improved treatment accessibility.^3^ Although progress has been made, challenges persist in improving patient outcomes.^1,3^

The WHO End TB Strategy recommends a target of ≥90% treatment success to reduce TB transmission, morbidity, and mortality.^4^ In Indonesia, the TB treatment success rate in 2022 was 86% overall but only 51% for MDR-TB patients.^3^ Factors previously associated with unfavourable outcomes in Indonesia include age ≥38 years, retreatment, cavitary pulmonary lesions, resistance to ≥1 anti-TB drugs, positive sputum smear at 2–3 months, HIV infection, and chronic kidney disease.^5,6^ Since most studies only involved single sites, a larger multisite study across Indonesia may identify more generalisable risk factors for unfavourable outcomes.

We analysed data on TB treatment from the Tuberculosis Research of INA-RESPOND on Drug Resistance (TRIPOD) study^7^ to identify factors associated with unfavourable treatment outcomes.

## METHODS

### Study setting, population and procedures

TRIPOD was a prospective observational cohort study of presumptive pulmonary TB adults conducted at seven TB referral hospitals in Indonesia from 2017 to 2020.^7^ Patients who were pregnant, had serious underlying conditions (e.g., chronic liver disease, chronic kidney disease, or psychiatric illness), or had TB treatment for ≥7 days in the 30 days before study enrolment were excluded. Baseline demographics, clinical data, and laboratory results (haemoglobin [Hb], glycated haemoglobin [HbA1c] and HIV) were collected. Body mass index (BMI) was categorised as underweight (<18.5 kg/m^2^), normal (18.5–24.9 kg/m^2^), or overweight (≥25 kg/m^2^),^8^ and anaemia was defined as Hb <13 g/dL for men and <12 g/dL for women.^9^ Participants were classified as diabetic according to medical history or HbA1c ≥6.5% at baseline.^10^

A minimum of 2 mL of sputum was collected for acid-fast bacilli (AFB) smear, Xpert MTB/RIF (Cepheid, Sunnyvale, CA, USA) test, *Mycobacterium tuberculosis* (MTB) culture, and phenotypic drug susceptibility testing (pDST). AFB smear-positive specimens were reported semi-quantitatively (scanty, 1+, 2+, 3+). AFB smear and Xpert MTB/RIF tests were performed at each hospital. MTB culture and pDST were performed at national TB referral laboratories, with sputum inoculated into Lowenstein-Jensen medium, Mycobacteria Growth Indicator Tubes or Ogawa medium. Drug concentrations used for pDST were 1.0 µg/ml rifampicin (RIF), 0.1 µg/ml isoniazid, 5.0 µg/ml ethambutol, 1.0 µg/ml streptomycin, 2.0 µg/ml ofloxacin, 1.0 µg/ml amikacin, and 1.0 µg/ml kanamycin.^11^

Participants were categorised as bacteriologically confirmed TB if sputum tested positive by Xpert MTB/RIF or MTB culture. This approach differed from WHO and national guidelines by excluding AFB smear-positive results due to their low sensitivity and specificity for MTB diagnosis. The classification, definitions, and management of DS-TB and DR-TB followed the 2016 Indonesian Ministry of Health Regulation on TB Control, which was active during the study period (Supplementary Tables S1 and S2).^12^ The time interval between TB diagnosis and treatment initiation was used to assess treatment delay, following the WHO definition,^13^ and was categorised as ≤7 days and >7 days, in line with national guidelines recommending treatment initiation within 7 days of diagnosis.^14^

Patients who tested positive for MTB by Xpert MTB/RIF received DS-TB treatment if the result demonstrated susceptibility to RIF, whereas those with RIF resistance were treated for MDR-TB. New DS-TB patients received DS-TB Category 1 treatment, while previously treated DS-TB patients received DS-TB Category 1 or 2 treatment. When TRIPOD began, only the longer MDR-TB treatment regimen was available. During September 2017, the shorter 9–12-month injectable-based MDR-TB treatment regimen became available nationally. The attending physician was informed of baseline pDST study results approximately 2 months after sputum collection, and the clinical decision was left to their discretion.

Study follow-up visits were conducted monthly for 6 months after treatment initiation, followed by every 2 months until the end of treatment if longer than 6 months. Sputum for MTB culture was collected at Months 1, 2, and the end of treatment. According to national guidelines, TB treatment outcome categories were treatment success (completed or cured), unfavourable (failed, death or loss to follow-up [LTFU]), and not evaluated (Supplementary Tables S1 and S2).^12^ An expert panel classified deaths as ‘probable TB-related death’ if there was a high likelihood of TB as the cause of death, ‘possible TB-related death’ if TB could have contributed to the death but other factors or uncertainties were present, ‘non-TB-related death,’ or ‘unknown.’

### Ethical approval

The Health Research Ethics Committee of the Indonesia National Institute of Health Research and Development, Jakarta, Indonesia, provided ethical approval for TRIPOD. Informed consent was obtained from all participants. The study is registered at ClinicalTrials.gov (NCT02758236).

### Data management and statistical analysis

Data were collected on case report forms, entered in OpenClinica (OpenClinica, LLC, Needham, MA, USA), and analysed using RStudio^®^ software v2024.09.0 (Posit Software, PBC, Boston, MA, USA). Descriptive statistics were used to summarise participant characteristics based on the TB treatment regimen received. Univariable and multivariable logistic regression assessed associations between independent variables and treatment outcomes. All odds ratios (ORs) and adjusted ORs (aORs) were reported with 95% confidence intervals (CIs). Independent variables with a *P*-value of ≤0.05 were considered significant.

## RESULTS

### Participants and TB treatment characteristics

**Figure 1.**
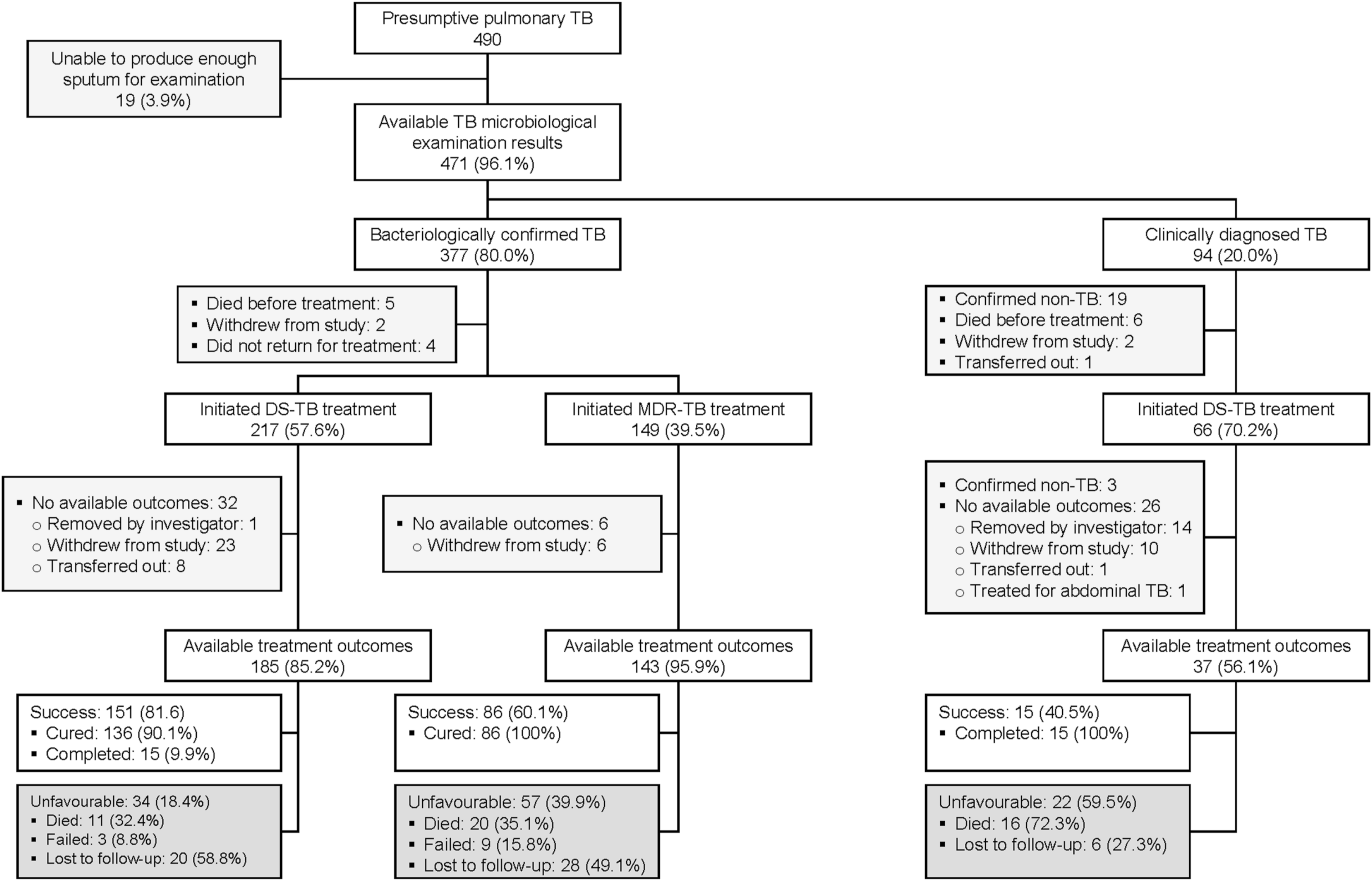
Flow of study participants from TB diagnosis to treatment to available outcomes. DS-TB = drug-susceptible TB; MDR-TB = multidrug-resistant TB.

Figure 1 presents the participant flow from TB diagnosis to treatment outcomes. Among 490 presumptive pulmonary TB patients enrolled, 377/471 (80.0%) had bacteriologically confirmed TB. Among 328 with available outcomes, 185 (56.4%) received DS-TB treatment, and 143 (43.6%) received MDR-TB treatment. Though the 94 participants with clinically diagnosed TB are not the focus of this analysis, 66 (70.2%) began TB treatment and 37 (56.1%) had available treatment outcomes, with 15 (40.5%) completing treatment, 16 (43.2%) died (Supplementary Tables S3), and 6 (16.3%) were lost to follow-up.

The baseline characteristics of participants with bacteriologically confirmed DS-TB and MDR-TB treatment with available outcomes are summarised in Table 1. In both treatment groups, over half of the participants were male, with median ages of 37 and 43 years, respectively. Most DS-TB treatment participants were new TB cases, whereas most MDR-TB treatment participants were previously treated. Participants treated for MDR-TB had more extensive pulmonary disease than participants treated for DS-TB. Treatment initiation after diagnosis was significantly longer for MDR-TB vs. DS-TB treatment participants (median 11 days, interquartile range [IQR] 3–20 vs. 4 days, IQR 1–7; *P* < 0.001). The median TB treatment completion duration among DS-TB treatment participants was 8.4 months (IQR 6.1–10.7). Of 125 participants completing DS-TB Category 1 treatment, only 32 (25.6%) completed within the recommended 6 months. Among the 93 (74.4%) participants with treatment durations ≥6 months, 10 (10.7%) had interruptions, with a median duration of interruption of 3 days (IQR 2–9). For MDR-TB treatment participants, 67.1% received the longer MDR-TB regimen with a median duration of 22.9 months (IQR 21.2–25.1), and 32.9% received the shorter injectable-based MDR-TB regimen with a median duration of 10.4 months (IQR 10.1–11.2).

**Table 1.** Characteristics of study participants with bacteriologically confirmed TB on DS-TB or MDR-TB treatment regimens and with available treatment outcomes.

Characteristics	Bacteriologically confirmed DS-TB treatment (*n* = 185)	Bacteriologically confirmed MDR-TB treatment (*n* = 143)
	*n* (%)	*n* (%)
Sex		
Male	117 (63.2)	82 (57.3)
Female	68 (36.8)	61 (42.7)
Age, years		
Median [IQR]	37 [24–50]	43 [36–52]
18–29	71 (38.4)	22 (15.4)
30–39	31 (16.8)	35 (24.5)
40–49	35 (18.9)	37 (25.9)
≥50	48 (25.9)	49 (34.3)
Current smoking status	34 (18.4)	9 (6.3)
TB treatment history		
New case	142 (76.8)	41 (28.7)
Previously treated	43 (23.2)	102 (71.3)
BMI, kg/m^2^		
Median [IQR]	18.4 [16.4–21.0]	18.7 [16.9–21.3]
<18.5 (underweight)	93 (50.3)	70 (49.0)
18.5–24.9 (normal)	79 (42.7)	64 (44.7)
≥25 (overweight)	13 (7.0)	9 (6.3)
Anaemia	101 (54.6)	62 (43.4)
HIV status		
Positive	7 (3.8)	0 (0.0)
Negative	175 (94.6)	142 (99.3)
Unknown	3 (1.6)	1 (0.7)
Known diabetes or HbA1C ≥6.5 at baseline	53 (28.6)	65 (45.5)
Lung lesions on chest X-ray		
≤2 zones	53 (28.6)	25 (17.5)
>2 zones	132 (71.4)	118 (82.5)
Presence of cavity	75 (40.5)	96 (67.1)
Presence of pleural effusion	32 (17.3)	46 (32.2)
Baseline AFB smear grade		
Negative	29 (15.7)	23 (16.1)
Scanty/+1	76 (41.1)	35 (24.5)
+2/+3	80 (43.2)	85 (59.4)
Baseline pDST results		
Unavailable	33 (17.8)	19 (13.3)
Available baseline pDST results	152 (82.2)	124 (86.7)
Drug susceptible	125/152 (82.2)	11/124 (8.9)
Mono-resistance	14/152 (9.2)	5/124 (4.0)
Poly-resistance	11/152 (7.2)	4/124 (3.2)
Rifampicin resistance or MDR-TB	2/152 (1.4)	93/124 (75.0)
Pre-XDR-TB or XDR-TB	0/152 (0.0)	11/124 (8.9)
TB treatment initiation after diagnosis		
Median [IQR]	4 [1–7]	11 [3–20]
≤7 days	139 (75.1)	57 (39.9)
>7 days	46 (24.9)	86 (60.1)
TB-treatment regimen		
DS Category 1	151 (81.6)	NA
DS Category 2	34 (18.4)	NA
Shorter injectable-based MDR-TB	NA	47 (32.9)
Longer MDR-TB	NA	96 (67.1)
MTB culture results at 2 months		
Negative MTB	129 (69.7)	96 (67.1)
Positive MTB	9 (4.9)	15 (10.5)
Unavailable	47 (25.4)	32 (22.4)
Not attending study visit Month 2	5/47 (10.6)	2/32 (6.3)
No sputum collection	34/47 (72.3)	3/32 (9.4)
Death or LTFU before Month 2	8/47 (17.0)	27/32 (84.4)

DS-TB = drug-susceptible TB; MDR-TB = multidrug-resistant TB; IQR = interquartile range; BMI = body mass index; HbA1C = glycated haemoglobin; AFB = acid-fast bacilli; pDST = phenotypic drug susceptibility testing; XDR-TB = extensively drug-resistant TB; NA = not applicable; MTB = *Mycobacterium tuberculosis*; LTFU = loss to follow-up.

Available pDST results essentially confirmed the baseline Xpert MTB/RIF results among participants treated for DS-TB, with only 2/152 (1.4%) isolates identified as RR-TB. 13/152 (8.6%) isolates were RIF-susceptible but isoniazid-resistant. Among participants treated for MDR-TB, 11/124 (8.9%) isolates were RIF-susceptible by pDST, which differed from the corresponding Xpert MTB/RIF results. Separately, 11/124 (8.9%) were classified as pre-extensively drug-resistant TB (pre-XDR-TB) or XDR according to the 2016 National TB Guidelines. The details of TB treatment regimens, durations, and outcomes as categorised by baseline pDST results are presented in Supplementary Figures S1 and S2.

Of the 293 participants followed until Month 2 of treatment, 249 (84.9%) had available sputum MTB culture results (Supplementary Figure S3 and Supplementary Table S4). Positive MTB culture results were found in 24/249 (9.6%) specimens and were more frequent in participants treated for MDR-TB than in those treated for DS-TB (10.5% vs. 4.9%).

### TB treatment outcomes

**Figure 2.**
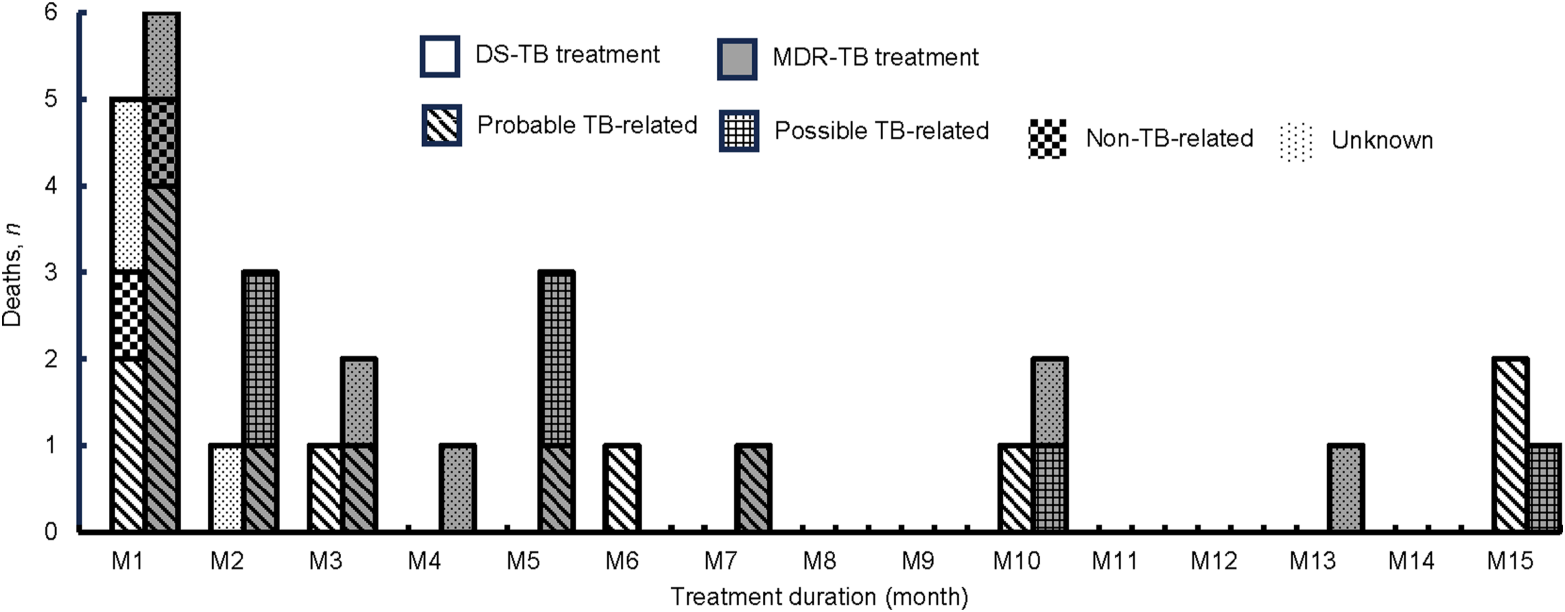
Distribution of 31 death cases categorised by TB treatment group and cause of death as it related to the TB infection. DS-TB = drug-susceptible TB; MDR-TB = multidrug-resistant TB.

Treatment success was observed in 72.3% (237/328) of participants: 81.6% (151/185) in DS-TB and 60.1% (86/143) in MDR-TB treatment groups (Figure 1). Unfavourable outcomes were observed in 27.7% (91/328): 3.6% (12/328) failures, 9.5% (31/328) deaths and 14.6% (48/328) LTFU. The proportion of failures, deaths, and LTFU among MDR-TB treatment participants (6.3%, 14.0% and 19.6%, respectively) was higher than DS-TB treatment participants (1.6%, 6.0% and 10.8%, respectively). The median duration from treatment initiation to LTFU was 3.5 months (IQR 1.9–4.7) in DS-TB and 1 month (IQR 0.5–2.4) in MDR-TB treatment participants. The median duration from treatment initiation to death was 1.5 months (IQR 0.9–8.2) in DS-TB and 2.8 months (IQR 0.7–5.9) in MDR-TB treatment participants. Figure 2 shows the distribution of the 31 deaths during treatment, of which 21 (67.7%) were probably or possibly TB-related.

### Factors associated with unfavourable outcomes in TB treatment

Table 2 presents factors associated with unfavourable treatment outcomes. Significant findings from a multivariable analysis included age ≥50 years (aOR 2.54, 95% CI 1.11–5.95; *P* = 0.029); being underweight (aOR 1.93, 95% CI 1.05–3.61; *P* = 0.037); having baseline AFB smear of scanty/+1 (aOR 3.77, 95% CI 1.41–11.65; *P* = 0.013) or +2/+3 (aOR 3.34, 95% CI 1.31–9.83; *P* = 0.017); and being treated with MDR-TB regimen (aOR 2.03, 95% CI 1.05–3.96; *P* = 0.036).

**Table 2.** Univariable and multivariable logistic regression analyses of factors associated with unfavourable outcomes among bacteriologically confirmed TB participants with available outcomes.*

Independent variable	Treatment success (*n* = 237) *n* (%)	Unfavourable outcomes (failure or death or LTFU) (*n* = 91) *n* (%)	Unadjusted analysis		Adjusted analysis	
			Unadjusted OR (95% CI)	*P*-value	Adjusted OR (95% CI)	*P*-value
Sex						
Male	143 (71.9)	56 (28.1)	—	—	—	—
Female	94 (72.9)	35 (27.1)	0.95 (0.58–1.56)	0.842	1.06 (0.60–1.86)	0.830
Age, years						
18-29	77 (82.8)	16 (17.2)	—	—	—	—
30-39	50 (75.8)	16 (24.2)	1.54 (0.70–3.38)	0.277	1.06 (0.44–2.55)	0.903
40-49	52 (72.2)	20 (27.8)	1.85 (0.88–3.95)	0.106	1.28 (0.53–3.09)	0.582
≥50	58 (59.8)	39 (40.2)	3.24 (1.67–6.49)	0.001	2.54 (1.11–5.95)	0.029
BMI, kg/m^2^						
Normal or overweight (≥18.5)	125 (75.8)	40 (24.2)	—	—	—	—
Underweight (<18.5)	112 (68.7)	51 (31.3)	1.42 (0.88–2.32)	0.155	1.93 (1.05–3.61)	0.037
TB treatment history						
New case	146 (79.8)	37 (20.2)	—	—	—	—
Previously treated	91 (62.8)	54 (37.2)	2.34 (1.43–3.86)	0.001	1.67 (0.90–3.11)	0.103
Anaemia						
No	123 (75.5)	40 (24.5)	—	—	—	—
Yes	114 (69.9)	49 (30.1)	1.32 (0.81–2.16)	0.264	1.27 (0.72–2.24)	0.406
Known diabetes or HbA1C ≥6.5 at baseline						
No	164 (78.1)	46 (21.9)	—	—	—	—
Yes	73 (61.9)	45 (38.1)	2.20 (1.34–3.61)	0.002	1.77 (0.91–3.48)	0.093
Lung lesions on chest X-ray						
≤2 zones	62 (79.5)	16 (20.5)	—	—	—	—
>2 zones	175 (70.0)	75 (30.0)	1.66 (0.92–3.15)	0.105	1.13 (0.57–2.31)	0.733
Presence of cavity						
No	123 (78.3)	34 (21.7)	—	—	—	—
Yes	114 (66.7)	57 (33.3)	1.81 (1.11–2.99)	0.019	1.17 (0.66–2.05)	0.593
Pleural effusion						
No	183 (73.2)	67 (26.8)	—	—	—	—
Yes	54 (69.2)	24 (30.8)	1.21 (0.69–2.10)	0.495	1.03 (0.54–1.93)	0.937
Baseline AFB smear grade						
Negative	45 (86.5)	7 (13.5)	—	—	—	—
Scanty/+1	82 (73.9)	29 (26.1)	2.27 (0.97–6.01)	0.074	3.77 (1.41–11.65)	0.013
+2/+3	110 (66.7)	55 (33.9)	3.21 (1.44–8.22)	0.008	3.34 (1.31–9.83)	0.017
TB treatment initiation after diagnosis						
≤7 days	149 (76.0)	47 (24.0)	—	—	—	—
>7 days	88 (66.7)	44 (33.3)	1.59 (0.97–2.59)	0.064	1.32 (0.75–2.33)	0.329
TB treatment regimen						
DS-TB	151 (81.6)	34 (18.4)	—	—	—	—
MDR-TB	86 (60.1)	57 (39.9)	2.94 (1.79–4.89)	<0.001	2.03 (1.05–3.96)	0.036

* Current smoking behaviour and HIV status were not included in the analysis due to small sample sizes.

LTFU = loss to follow-up; OR = odds ratio; CI = confidence interval; BMI = body mass index; HbA1C = glycated haemoglobin; AFB = acid-fast bacilli; DS-TB = drug-susceptible TB; MDR-TB = multidrug-resistant TB.

An additional univariable analysis was conducted to evaluate the association between MTB culture results at Month 2 and treatment outcomes. This variable was not included in the multivariable analysis, as that model focused on baseline predictors. In this univariable analysis, a positive MTB culture at Month 2 was significantly associated with unfavourable outcomes (OR 3.37, 95% CI 1.32–8.22; *P* = 0.008). Unavailable results were also associated with unfavourable outcomes (OR 2.36, 95% CI 1.09–4.89; *P* = 0.024) (Supplementary Table S5).

## DISCUSSION

In this analysis of 328 bacteriologically confirmed Indonesian TB participants with available outcomes, 237 (72.3%) were successfully treated, including 151 (81.6%) DS-TB and 86 (60.1%) MDR-TB participants. Unfavourable outcomes occurred in 91 (27.7%), with treatment failure in 12 (3.6%), death in 31 (9.5%), and LTFU in 48 (14.6%). MDR-TB participants had a higher proportion of unfavourable outcomes (57/143, 39.9%) compared to DS-TB participants (34/185, 18.4%). Factors significantly associated with unfavourable outcomes included age ≥50 years, being underweight, having a baseline positive AFB smear, and being treated with the MDR-TB regimen.

The WHO stresses the importance of microbiological detection for accurate TB diagnosis and appropriate treatment.^1^ Our study supports national policies expanding free Xpert MTB/RIF testing to all presumptive TB patients, and the WHO End TB Strategy calls for universal access to DST using molecular and phenotypic methods to assess resistance patterns.^15^ As observed in our study, discordant rifampicin results between Xpert MTB/RIF and pDST may be attributed to Xpert’s detection of low-level resistance or silent mutations, technical issues, or mixed infections with both susceptible and resistant strains.^11^ Sequencing or determining minimum inhibitory concentrations of anti-TB drugs for confirmation may assist the treatment decision.^16^

The time interval from diagnosis to treatment initiation was longer among MDR-TB treatment compared to DS-TB treatment (median 4 vs. 11 days). Efforts should focus on expanding, decentralising, and improving care to reduce treatment delay.^17^ Extended treatment duration in DS-TB treatment without reported adherence issues was common in our study. Reasons for treatment extension included cavitation, persistent AFB smear or culture positivity, adverse drug events, comorbidities (e.g., HIV, diabetes), extrapulmonary TB, and slow clinical or radiological improvement.^18^ Future research is needed to assess the benefits and disadvantages of this approach.

We observed an overall success of 72.3% in our study, below the WHO target of 90%.^4^ The 2021 national TB report for Indonesia showed treatment success from 72.1% to 96.2% across 34 provinces, with only nine provinces meeting the target.^3^ Primary health care achieved 90% treatment success for DS-TB, while government hospitals managing more complex cases of both DS and MDR-TB had a rate of 79%.^3^ The high proportion of LTFU observed in our study represents a significant obstacle in TB control; however, reasons for LTFU were not assessed. A study in Bandung, Indonesia, reported that LTFU was associated with liver disease, severe underweight, unresolved symptoms, socio-economic issues, and low healthcare satisfaction.^19^

Almost half of the possible or probable TB-related deaths in this study occurred within the first 2 months of treatment initiation, likely due to extensive lung damage, high bacterial load, and immunopathologic response.^20^ The severity of cases may reflect our study sites as referral hospitals handling complex cases, often with diagnosis and treatment delays. Additionally, factors such as malnutrition, diabetes, anaemia, and drug resistance likely reduced treatment effectiveness.^21,22^ Timely anti-TB treatment, appropriate medical intervention, and management of co-existing diseases are crucial to reducing mortality.

In this analysis, individuals aged ≥50 years were found to have a higher risk of unfavourable outcomes than those aged 18–29 years. Older age has been identified elsewhere as a risk factor due to immunosenescence, comorbidities, poor drug absorption, frequent adverse events, and socio-economic challenges.^5,23^ As Indonesia transitions toward an ageing population,^24^ this high-risk group requires special attention and may benefit from tailored, multidisciplinary management and social support. As reported in other studies, being underweight was significantly associated with unfavourable outcomes.^25,26^ Therefore, nutritional assessment and intervention, including dietary programmes, counselling, ensuring food security, and maintaining appropriate micronutrient supplementation, may improve outcomes.^27,28^

Baseline positive AFB smear was associated with unfavourable outcomes, possibly due to high bacillary burden leading to incomplete eradication.^29^ The inverted association between higher AFB grades and aORs may be due to the smaller sample size of the scanty/+1 group. Despite mixed evidence of Month 2 MTB culture results as an outcome predictor,^30^ we acknowledge its frequent use in treatment monitoring. Close and comprehensive monitoring, including culture and clinical assessments, can aid in evaluating disease progression and guiding treatment.

The association of MDR-TB treatment regimens with unfavourable outcomes aligns with previous publications.^31,32^ MDR-TB strains are more challenging to treat than DS-TB strains, requiring second-line anti-TB regimens with higher rates of adverse events and treatment interruption.^32,33^ MDR-TB management is also complicated by programmatic factors like funding, drug supply challenges, and limited MDR-TB care and laboratory services, along with patient factors such as extensive lung damage, comorbidities, and psychosocial-economic issues.^33^ Notably, during the study, patients treated for MDR-TB received the conventional 20–24 months or the shortened 9–12 months injectable-based treatment. Since 2022, Indonesia has gradually expanded the WHO-recommended 6-month all-oral regimen with bedaquiline, pretomanid, linezolid, and moxifloxacin (BPaLM) programmatically.^3^ It is worth assessing how outcomes may have improved following the introduction of 6-month all-oral MDR-TB regimens in Indonesia.

This analysis has some limitations. First, the treatment outcomes observed may not represent TB across Indonesia, as participants were enrolled from referral hospitals and likely presented with more severe disease. Second, our study did not assess socio-economic status, adherence, adverse drug events, and MTB strains, which may be associated with outcomes.

## CONCLUSION

The overall treatment success of 72.3% is below the WHO target of 90%, emphasising the need for improvements. We recommend strategies based on factors associated with unfavourable outcomes identified in our study. First, comprehensive and tailored management is needed for older patients. Second, it is essential to assess nutritional status before treatment, followed by targeted interventions and monitoring. Third, pre-treatment positive AFB smears should be closely monitored with proper follow-up. Finally, improving care for MDR-TB patients is crucial due to the complexities of drug-resistant infections and various patient and programmatic challenges.

## Supplementary Material


