# Supplementary material for: Treatment outcomes of pulmonary TB in adults in Indonesia
Source: IJTLD Open. 2025 Mar 12;2(3):145–52. doi: 10.5588/ijtldopen.24.0482 (PMC11906029; doi:10.5588/ijtldopen.24.0482)
Supplement: Supplementary file 1 [file ijtldopen24-0482_supplementarydata1.pdf]

http://dx.doi.org/10.5588/ijtldopen.24.0482

Treatment outcomes of pulmonary TB in adults in Indonesia

SUPPLEMENTARY DATA

Supplementary Table S1. Operational definitions based on the 2016 Indonesian Ministry of Health tuberculosis control regulation.

Terms	Definition
Drug-susceptible TB (DS-TB)	TB case without evidence of resistant to anti-TB drugs.
Rifampicin resistant TB (RR-TB)	TB case with evidence of resistant to rifampicin, with or without resistant to other anti-TB drugs, based on DST or molecular results. RR-TB is eligible for treatment with MDR-TB regimens.
Multi-drug resistant TB (MDR-TB)	TB case with evidence of resistant to rifampicin and isoniazid.
Mono-resistant TB (Mono-R-TB)	TB case with evidence of resistant to one of 1st line anti-TB drug other than rifampicin.
Polydrug resistant TB (Poly-R-TB)	TB case with evidence of resistant to more than one of 1st line anti-TB drugs, other than combination of rifampicin and isoniazid.
Pre-Extensively drug resistant TB (pre-XDR-TB)	MDR-TB case with evidence of resistant to any fluoroquinolone or one of three second-line injectable drugs (amikacin, kanamycin, or capreomycin).
Extensively drug resistant TB (XDR-TB)	MDR-TB case with evidence of resistant to any fluoroquinolone and to at least one of three second-line injectable drugs.
Cured	DS-TB: A TB patient who was bacteriologically confirmed at the start of treatment and is AFB smear or culture-negative at the end of treatment, as well as at least one previous examination.<br>MDR-TB: A TB patient who completes treatment without evidence of failure, with ≥3 consecutive negative cultures taken at least 30 days apart during the continuation phase.
Completed	DS-TB: A TB patient who completes treatment with at least one negative AFB smear or culture before the end of treatment but without evidence of negative results at the end of treatment.<br>MDR-TB: A TB patient who completes treatment but has inadequate bacteriologic records to be classified as cured.
Failure	DS-TB: A TB patient whose sputum AFB smear or culture is still positive or bacteriologically reverted at the fifth month or later during the treatment.<br>MDR-TB: A TB patient whose treatment is terminated or needs for permanent regimen change at least two anti-TB drugs because of:<br>• No conversion at the end of intensive phase; or<br>• Bacteriological reversion in the continuation phase; or<br>• Evidence of additional acquired resistance to fluoroquinolones or second-line injectable drugs; or severe adverse drug reactions
Died	A TB patient who dies from any cause during the treatment.
Lost-to follow up	A TB patient who interrupts the treatment for eight or more consecutive weeks.
Not evaluated	A TB patient for whom no treatment outcome is assigned. This includes cases “transferred out” to another treatment unit and where the treatment outcome is unknown.

Reference: Ministry of Health Republic of Indonesia. The Ministry of Health of the Republic of Indonesia regulation No.67 years 2016 on tuberculosis control [Peraturan Menteri Kesehatan Republik Indonesia No. 67 tahun 2016 tentang penanggulangan tuberkulosis]. 2016.

Supplementary Table S2. National tuberculosis treatment recommendations based on the 2016 Indonesian Ministry of Health tuberculosis control regulation.

Regimen	National Recommendation of Anti-TB Drugs Combination and Duration (2016)
DS-TB Category 1	Two months of intensive phase: RHZE<br>Four months of continuation phase: RH
DS-TB Category 2	Three months of intensive phase: (RHZE)S for two months and RHZE for one month<br>Five months of continuation phase: RHE
Longer MDR-TB	At least five effective TB medicines during the intensive phase (four months after culture conversion, at least eight months), including pyrazinamide and four core second-line TB medicines – one chosen from Group A, one from Group B, and at least two from Group C2. If the minimum number of effective TB medicines cannot be composed as given above, an agent from Group D2 and other agents from Group D3 may be added to bring the total to five.<br>12-14 months of continuation phase
Shorter injectable based MDR-TB	Four-six months of intensive phase: Km-Mfx-Eto-high H-Cfz-Z<br>Five months of continuation phase: Mfx-Eto-Cfz-Z

TB = tuberculosis; DS = drug-susceptible; MDR: multidrug-resistant; R = Rifampicin; H = Isoniazid; Z = Pyrazinamide; E = Ethambutol; S = Streptomycin; Km = Kanamycin; Eto = Ethionamide; Mfx = Moxifloxacin; Cfz = Clofazimine.

Group A=levofloxacin, moxifloxacin, gatifloxacin; Group B=amikacin, capreomycin, kanamycin, streptomycin; Group C= ethionamide (or prothionamide), cycloserine (or terizidone), linezolid, clofazimine; Group D2=bedaquiline, delamanid; Group D3=p-aminosalicylic acid, imipenem–cilastatin, meropenem, amoxicillin clavulanate.

Reference: Ministry of Health Republic of Indonesia. The Ministry of Health of the Republic of Indonesia regulation No.67 years 2016 on tuberculosis control [Peraturan Menteri Kesehatan Republik Indonesia No. 67 tahun 2016 tentang penanggulangan tuberkulosis]. 2016.

Supplementary Table S3. Characteristics of death cases among clinically diagnosed tuberculosis participants.

Case, hospital city, gender, age (year)	TB treatment history	BMI (kg/m^2)	Haemo globin (g/dL)	Chest X-ray	HIV status	Comorbidities	Time from treatment initiation to death (days)	Clinical information before death
#01, Denpasar, male, 50	Previously-treated	24,2	12	Lung lesions >2 zones, no cavity, no pleural effusion	Unknown	-	14	Septic shock
#02, Denpasar, female, 31	New case	21,2	7,6	Lung lesions 2 zones, no cavity, pleural effusion	Unknown	-	4	No information
#03, Surabaya, female, 52	Previously-treated	21,2	13,2	Lung lesion 1 zone, no cavity, pleural effusion	Negative	-	38	Respiratory failure
#04, Yogyakarta, female, 26	New case	24,4	7,6	Lung lesions >2 zones, no cavity, no pleural effusion	Negative	Acute myeloid leukaemia, diabetes	6	Respiratory failure
#05, Yogyakarta, male, 31	New case	22,5	14,3	Lung lesions >2 zones, no cavity, no pleural effusion	Positive	-	34	No information
#06, Yogyakarta, male, 24	New case	19,0	7,1	Lung lesions >2 zones, no cavity, no pleural effusion	Positive	-	16	Septic shock
#07, Yogyakarta, male, 60	New case	22,0	13,4	Lung lesions >2 zones, no cavity, no pleural effusion	Negative	Osteochondroma	18	No information
#08, Yogyakarta, female, 46	New case	16,0	11,1	Lung lesions >2 zones, no cavity, no pleural effusion	Positive	-	24	Intracranial infection
#09, Yogyakarta, male, 49	Previously-treated	13,7	12,9	Lung lesions >2 zones, no cavity, pleural effusion	Negative	-	33	Pneumonia
#10, Yogyakarta, female, 36	New case	20,0	9,0	Lung lesions 2 zones, no cavity, no pleural effusion	Unknown	-	42	Sepsis

#11, Yogyakarta, female, 27	New case	15,2	5,5	Lung lesions >2 zones, no cavity, no pleural effusion	Positive	-	10	Cerebral toxoplasmosis
#12, Yogyakarta, male, 32	Previously-treated	17,0	8,5	Lung lesions >2 zones, no cavity, no pleural effusion	Positive	-	2	Septic shock
#13, Yogyakarta, female, 64	New case	23,4	7,9	Lung lesions >2 zones, no cavity, no pleural effusion	Negative	Acute lymphocytic leukaemia, histoplasmosis	5	Septic shock
#14, Yogyakarta, male, 34	New case	20,7	10,1	Lung lesions >2 zones, no cavity, no pleural effusion	Negative	Pulmonary mass, Diabetes	10	Pulmonary embolism
#15, Jakarta, male, 24	New case	15,8	13,1	Lung lesions >2 zones, no cavity, no pleural effusion	Positive	-	17	Sepsis
#16, Medan, male, 55	New case	19,5	14,1	Lung lesions 2 zones, no cavity, no pleural effusion	Negative	-	3	No information

TB = tuberculosis; BMI = body mass index; HIV = human immunodeficiency virus. A high death rate was observed among clinically diagnosed TB participants (16/37, 43.2%), potentially due to coinfections (e.g., HIV or other pathogens) and comorbidities. Among the 16 death cases in clinically diagnosed TB, 4 (25%) were underweight, 11 (68.8%) had anaemia (5 of 11 (45.5%) with severe anaemia), and 12 (75%) had lung lesions >2 zones. HIV co-infection was observed in 6 (37.5%) cases, and 4 (25%) had underlying malignancies. The median (IQR) time from TB treatment initiation to death was 15 (6–26) days. In 6 (37.5%) cases, sepsis preceded death.

Supplementary Table S4. Characteristics of study participants with bacteriologically confirmed tuberculosis and available outcomes according to the availability of MTB culture results at month 2.

Characteristics	Available MTB culture results at month 2 (N=249) *n (%)*	Unavailable MTB culture results at month 2 (N=44) *n (%)*	p-value
Gender			
Male	145 (58.2)	36 (81.8)	0.005
Female	104 (41.8)	8 (18.2)	
Age (years)			
18-29	77 (30.9)	12 (27.3)	0.815
30-39	52 (20.9)	10 (22.7)	
40-49	55 (22.1)	8 (18.2)	
≥50	65 (26.1)	14 (31.8)	
Current smoking status	25 (10.0)	14 (31.8)	<0.001
TB treatment history			
New case	138 (55.4)	29 (65.9)	0.258
Previously treated	111 (44.6)	15 (34.1)	
BMI (kg/m^2)			
<18.5 (underweight)	128 (51.4)	22 (50.0)	0.993
18.5-24.9 (normal) or ≥25 (overweight)	121 (48.6)	22 (50.0)	
Anaemia	122 (49.0)	25 (56.8)	0.442
HIV status			
Positive	4 (1.6)	3 (6.8)	0.106
Negative	244 (98.0)	39 (88.6)	
Unknown	1 (0.4)	2 (4.5)	
Known diabetes or HbA1C ≥ 6.5 at baseline	83 (33.3)	13 (29.5)	0.750
Lung lesions on chest X-ray			
≤2 zones	65 (26.1)	5 (11.4)	0.055
>2 zones	184 (73.9)	39 (88.6)	
Presence of cavity	126 (50.6)	24 (54.5)	0.750
Presence of pleural effusion	57 (22.9)	15 (34.1)	0.161
Baseline AFB smear grade			
Negative	39 (15.7)	9 (20.5)	0.007
Scanty / +1	79 (31.7)	23 (52.3)	
+2/+3	131 (52.6)	12 (27.3)	
TB-treatment delays (days)			
≤7 days	147 (59.0)	32 (72.7)	0.121
>7 days	102 (41.0)	12 (27.3)	
TB-treatment regimen			
DS-TB	138 (55.4)	39 (88.6)	<0.001
MDR-TB	111 (44.6)	5 (11.4)	

MTB = Mycobacterium tuberculosis; TB = tuberculosis; BMI = body mass index; HIV = human immunodeficiency virus; AFB = acid-fast bacilli; DS = drug-susceptible; MDR: multidrug-resistant. The availability of culture results at month 2 appears to be influenced by factors such as gender (a higher proportion of males with unavailable culture results), current smoking status (higher among those without culture results), baseline AFB smear grade (more frequently +2/+3 in those with available culture results), and TB treatment regimen (a higher proportion of DS-TB treatment in the unavailable culture results group compared to MDR-TB treatment). In our study, participants without culture results at month 2 may have missed follow-up visits, had low sputum production, experienced critical illness that prevented sputum collection, or faced technical issues in specimen collection and management.

Supplementary Table S5. Univariable logistic regression analysis of MTB culture results associated with unfavourable outcomes among active participants at month 2 treatment.

Independent variable	Treatment Success (N=237) n (%)	Unfavourable Outcomes (failure or death or LTFU) (N=56) n (%)	Unadjusted analysis	
			Unadjusted OR (95% CI)	p-value
MTB culture results at month 2 treatment				
Negative	191 (84.9)	34 (15.1)	-	-
Positive	15 (62.5)	9 (37.5)	3.37 (1.32-8.22)	0.008
Unavailable	31 (70.5)	13 (29.5)	2.36 (1.09-4.89)	0.024

LTFU = lost to follow-up; OR = odds ratio; CI = confidence interval; MTB = Mycobacterium tuberculosis.

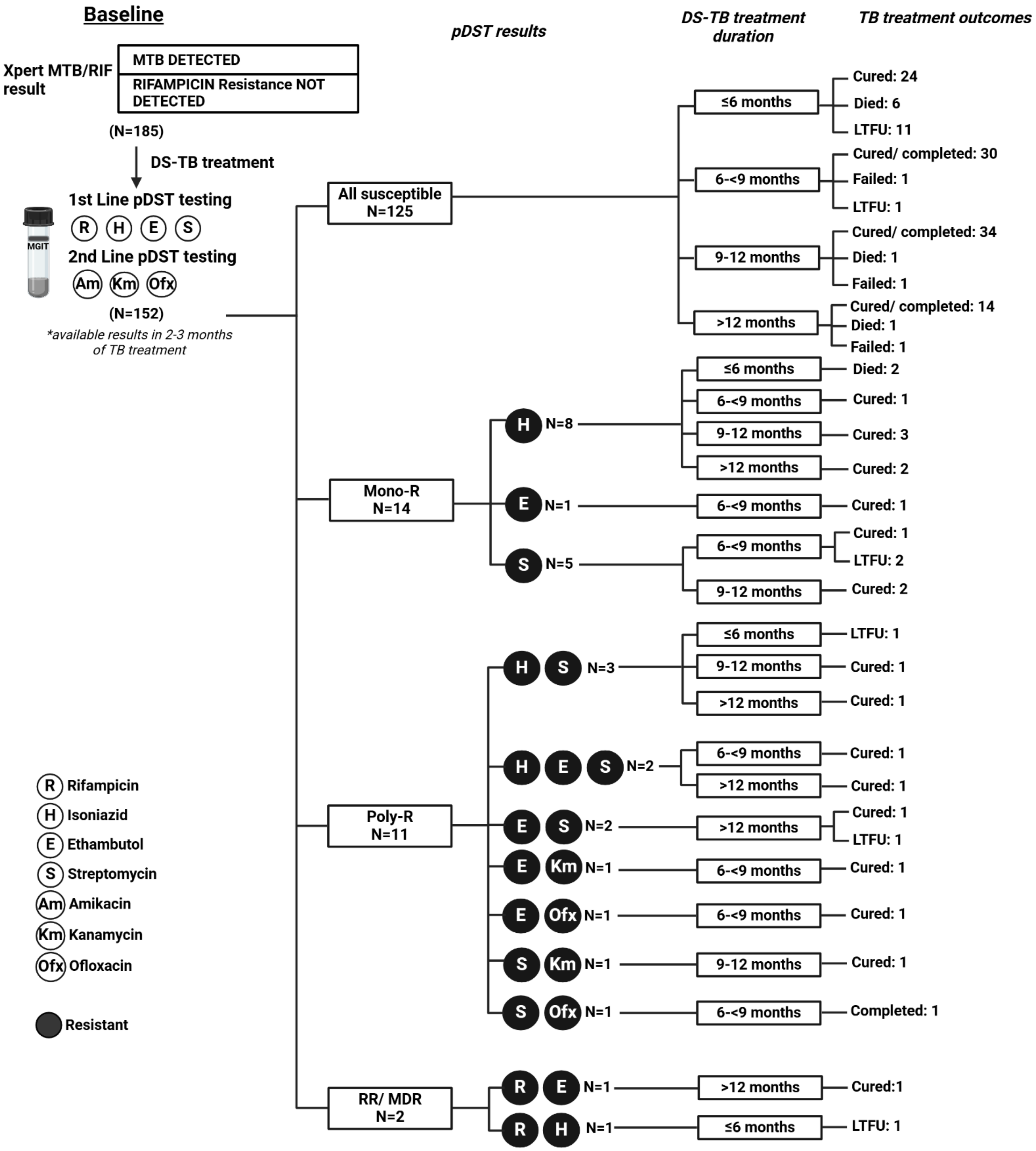

Supplementary Figure S1. Tuberculosis treatment duration and outcomes categorized based on baseline pDST results in DS-TB treatment. *MTB = Mycobacterium tuberculosis; TB = tuberculosis; pDST = phenotypic drug susceptibility testing; DS = drug-susceptible; Mono-R = mono-resistance; Poly-R = poly-resistance; RR = rifampicin-resistance.*

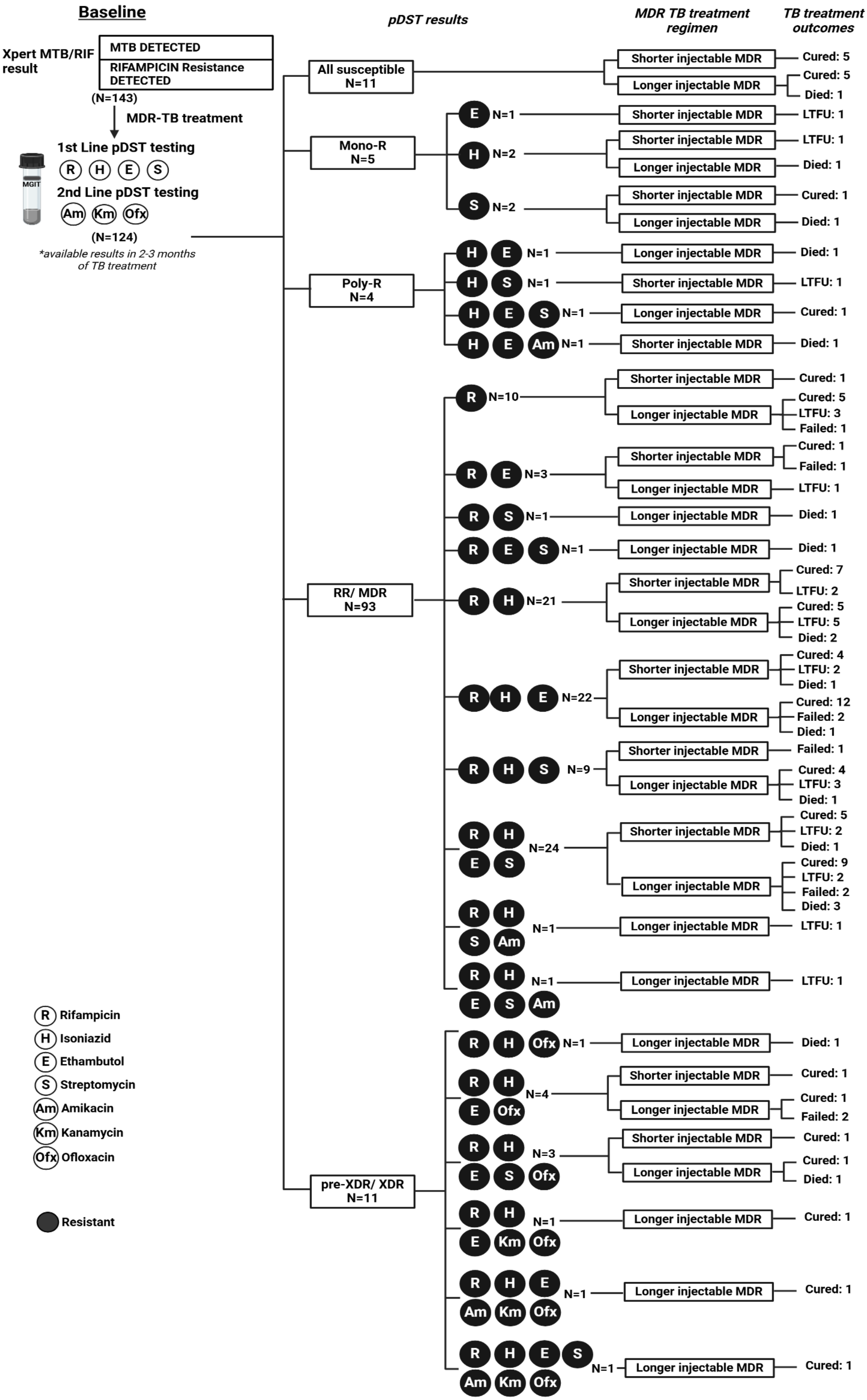

Baseline
pDST results
MDR TB treatment regimen
TB treatment outcomes
Xpert MTB/RIF result
MTB DETECTED
RIFAMPICIN Resistance DETECTED
(N=143)
MDR-TB treatment
1st Line pDST testing
R H E S
2nd Line pDST testing
Am Km Ofx
MGIT
(N=124)
*available results in 2-3 months of TB treatment
All susceptible N=11
Shorter injectable MDR
Cured: 5
Longer injectable MDR
Cured: 5
Died: 1
Mono-R N=5
E N=1
Shorter injectable MDR
LTFU: 1
H N=2
Shorter injectable MDR
LTFU: 1
Longer injectable MDR
Died: 1
S N=2
Shorter injectable MDR
Cured: 1
Longer injectable MDR
Died: 1
Poly-R N=4
H E N=1
Longer injectable MDR
Died: 1
H S N=1
Shorter injectable MDR
LTFU: 1
H E S N=1
Longer injectable MDR
Cured: 1
H E Am N=1
Shorter injectable MDR
Died: 1
RR/ MDR N=93
R N=10
Shorter injectable MDR
Cured: 1
Longer injectable MDR
Cured: 5
LTFU: 3
Failed: 1
R E N=3
Shorter injectable MDR
Cured: 1
Failed: 1
Longer injectable MDR
LTFU: 1
R S N=1
Longer injectable MDR
Died: 1
R E S N=1
Longer injectable MDR
Died: 1
R H N=21
Shorter injectable MDR
Cured: 7
LTFU: 2
Longer injectable MDR
Cured: 5
LTFU: 5
Died: 2
R H E N=22
Shorter injectable MDR
Cured: 4
LTFU: 2
Died: 1
Longer injectable MDR
Cured: 12
Failed: 2
Died: 1
R H S N=9
Shorter injectable MDR
Failed: 1
Longer injectable MDR
Cured: 4
LTFU: 3
Died: 1
R H E S N=24
Shorter injectable MDR
Cured: 5
LTFU: 2
Died: 1
Longer injectable MDR
Cured: 9
LTFU: 2
Failed: 2
Died: 3
R H S Am N=1
Longer injectable MDR
LTFU: 1
R H E S Am N=1
Longer injectable MDR
LTFU: 1
pre-XDR/ XDR N=11
R H Ofx N=1
Longer injectable MDR
Died: 1
R H E Ofx N=4
Shorter injectable MDR
Cured: 1
Longer injectable MDR
Cured: 1
Failed: 2
R H E S Ofx N=3
Shorter injectable MDR
Cured: 1
Longer injectable MDR
Cured: 1
Died: 1
R H E Km Ofx N=1
Longer injectable MDR
Cured: 1
R H E Am Km Ofx N=1
Longer injectable MDR
Cured: 1
R H E S Am Km Ofx N=1
Longer injectable MDR
Cured: 1
R Rifampicin
H Isoniazid
E Ethambutol
S Streptomycin
Am Amikacin
Km Kanamycin
Ofx Ofloxacin
Resistant

Supplementary Figure S2. Tuberculosis treatment regimens and outcomes categorized based on baseline pDST results in MDR-TB treatment. *MTB = Mycobacterium tuberculosis; TB = tuberculosis; pDST = phenotypic drug susceptibility testing; Mono-R = mono-resistance; Poly-R = poly-resistance; RR = rifampicin-resistance; MDR = multidrug-resistant; XDR = extensively drug-resistance.*

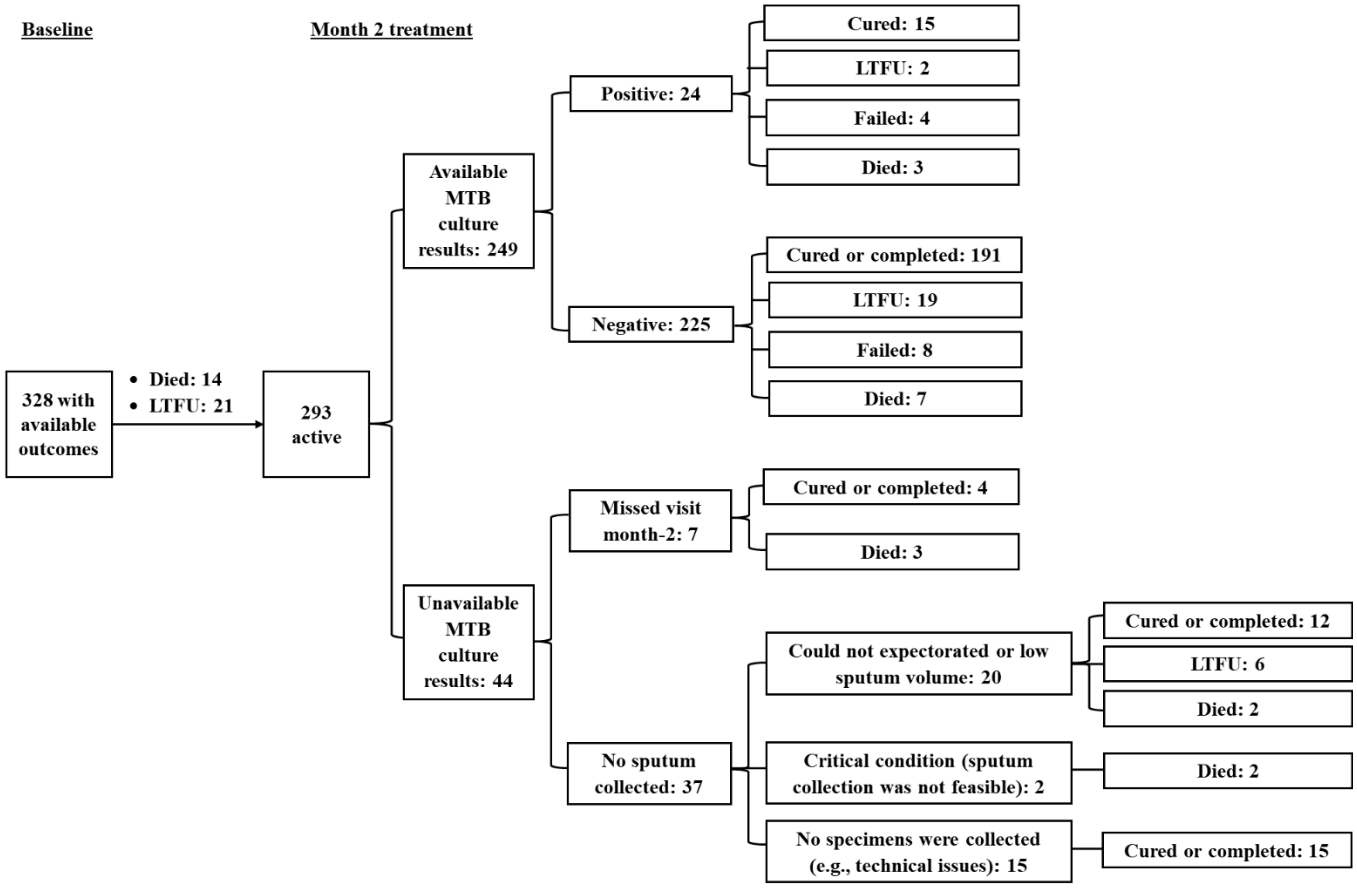

Supplementary Figure S3. Details on the availability of MTB culture results at month 2 of treatment, including information on unavailable results to treatment outcomes. *LTFU = lost to follow-up; MTB = Mycobacterium tuberculosis.*
